# Screening and Molecular Docking of Bioactive Metabolites of the Red Sea Sponge *Callyspongia siphonella* as Potential Antimicrobial Agents

**DOI:** 10.3390/antibiotics11121682

**Published:** 2022-11-23

**Authors:** Arafa Musa, Mohamed A. Abdelgawad, Mohamed E. Shaker, Ahmed H. El-Ghorab, Della Grace Thomas Parambi, Ahmed A. Hamed, Ahmed M. Sayed, Hossam M. Hassan, Mahmoud A. Aboseada

**Affiliations:** 1 Department of Pharmacognosy, College of Pharmacy, Jouf University, Sakaka 72341, Aljouf, Saudi Arabia; 2 Department of Pharmaceutical Chemistry, College of Pharmacy, Jouf University, Sakaka 72341, Aljouf, Saudi Arabia; 3 Department of Pharmacology, College of Pharmacy, Jouf University, Sakaka 72341, Aljouf, Saudi Arabia; 4 Department of Chemistry, College of Science, Jouf University, Sakaka 72341, Aljouf, Saudi Arabia; 5 National Research Centre, Microbial Chemistry Department, 33 El-Buhouth Street, Dokki, Giza 12622, Egypt; 6 Department of Pharmacognosy, Faculty of Pharmacy, Nahda University, Beni-Suef 62513, Egypt; 7 Department of Pharmacognosy, Faculty of Pharmacy, Beni-Suef University, Beni-Suef 62513, Egypt

**Keywords:** marine sponges, *Callyspongia siphonella*, anti-biofilm, antimicrobial, molecular docking, isolation, metabolites, pharmacophore-based docking

## Abstract

Marine sponges create a wide range of bioactive secondary metabolites, as documented throughout the year. Several bioactive secondary metabolites were isolated from different members of *Callyspongia siphonella* species. This study aimed for isolation and structural elucidation of major metabolites in order to investigate their diverse bioactivities such as antimicrobial and anti-biofilm activities. Afterwards, a molecular docking study was conducted, searching for the possible mechanistic pathway of the most bioactive metabolites. Extraction, fractionation, and metabolomics analysis of different fractions was performed in order to obtain complete chemical profile. Moreover, in vitro assessment of different bioactivities was performed, using recent techniques. Additionally, purification, structural elucidation of high features using recent chromatographic and spectroscopic techniques was established. Finally, AutoDock Vina software was used for the Pharmacophore-based docking-based analysis. As a result, DCM (dichloromethane) fraction exerted the best antibacterial activity using disc diffusion method; particularly against *S. aureus* with an inhibition zone of 6.6 mm. Compound **11** displayed a considerable activity against both MRSA (Methicillin-resistant *Staphyllococcus aureus*) and *Staphyllococcus aureus* with inhibition ratios of 50.37 and 60.90%, respectively. Concerning anti-biofilm activity, compounds **1** and **2** displayed powerful activity with inhibition ratios ranging from 39.37% to 70.98%. Pharmacophore-based docking-based analysis suggested elongation factor G (EF-G) to be a probable target for compound **11** (siphonellinol C) that showed the best *in vitro* antibacterial activity, offering unexplored potential for new drugs and treatment candidates.

## 1. Introduction

Marine sponges have recently been regarded as a very promising scope for the discovery of bioactive natural chemical substances pertaining to their primary and secondary metabolites diversity [1]. Numerous marine sponges regularly maintain a remarkable lack of habitation by tiny creatures and plants, as is well known (fouling organisms). It has been proposed that they accumulate physiologically active chemicals that prevent fouling organisms from settling, and thus being able to protect ships hulls, bridges and aquaculture materials from serious problems [2]. Furthermore, the diffusion of these chemicals in the tissues of marine sponges may improve the efficacy of the retention processes involved. Additionally, it might operate as a barrier against microbial diseases or as a tool for managing symbiotic bacteria populations [3]. The genus *Callyspongia* belongs to the family *Callyspongiidae*, order *Haplosclerida*. *Callyspongia siphonella* (Levi, 1995), sometimes known as colonial tube-sponge, is a species of Red Marine sea sponge [4]. A plethora of bioactive anticancer secondary metabolites, such as polyacetylenic alcohols, amides, sterols, cardenolides, peptides and sipholane triterpenes, have been discovered in this genus [5,6,7,8]. In addition, a variety of steroidal anti-inflammatory sterols such as callysterol have also been discovered from this genus [9]. Gram-positive and -negative bacterial biofilms have the potential to cause serious infections, especially in elderly and immunocompromised individuals. Unfortunately, currently available antibiotic remedies are ineffective in treating such infections [10,11]. Various *Callyspongia* crude extracts effectively countered a range of hazardous bacterial strains through their antibacterial and anti-biofilm characteristics [12].

In the ongoing work, bioactivity-guided fractionation accompanied by LC-HRESIMS of the Red Sea sponge *C. siphonella* resulted in the isolation of five known metabolites (**1**–**3**, **10**, and **11**). Compound **11** showed considerable antibacterial activity in both MRSA and *S. aureus*. Moreover, a docking study was applied to explore the possible mechanistic pathway of compound **11**.

## 2. Results and Discussion

### 2.1. Metabolomic Profiling

**Figure 1:**
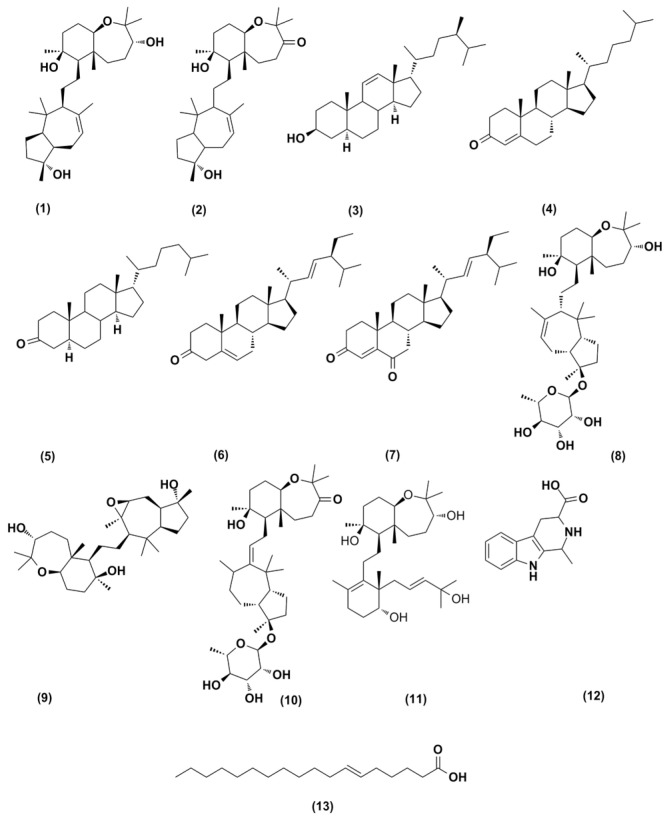
Identified compounds of *C. siphonella* DCM fraction by dereplication with LC-HRESIMS.

The mass resolution in this ongoing study was 50,000 (at *m*/*z* 400), which is sufficient to distinguish closely related compounds. Table 1 lists all the features that were detected by LC-HRMS (liquid chromatography coupled with high resolution mass spectrometry) concerning the *C. siphonella* DCM fraction, and the highest numbers of features were detected in the same fraction as documented in Figure 1, Table 2. The DCM fraction of *C. siphonella* was the most active one concerning the antimicrobial activity screening in the target-based functional assay on Gram-positive bacteria, Gram-negative bacteria, yeast, and fungi tested. The majority of the DCM fraction’s metabolites were ostensibly identified as triterpenes. Several of those were dereplicated as sipholanes which have already been described from this sponge [13,14,15]. Other plausible congeners were detected- for example steroids such as callysterol and stigmasterone [9,16], and amino acid analuoges such as 1,2,3,4-tetrahydro-1-methyl-β-carboline-3-carboxylic acid and fatty acids, namely petroselenic acid [17,18,19]. Many metabolites with high intensities were found, particularly triterpenes (sipholanes), by applying an algorithm to the data from this fraction to calculate the intensity of each *m*/*z* of parent ion peaks; see Table 2. For our knowledge, *C. siphonella* has yielded an increasing number of secondary metabolites with various pharmacological properties [20]. The principal distinctive metabolites of this sponge are sipholane triterpenoids. This family of compounds was discovered to be effective at reversing multidrug resistance in tumour cells overexpressing P-glycoprotein (P-gp) [21]. As a result, metabolomic profiling of the DCM fraction of sea sponge *C. siphonella* employing LC-HRESIMS for dereplication purposes resulted in the characterisation of a number of metabolites (Figure 1, Table 1 and Table 2), the most common of which were sipholane triterpenes. Moreover, the characteristic sterol of *C. siphonella* called callysterol (**3**) was reported to demonstrate in vitro anti-inflammatory activity [9]. Additionally, the cytotoxic steroids, stigmasterone [22] and stigmasta-4,22-dien-3,6-dione [23], in addition to one cytotoxic and antioxidant amino acid analogue, 1,2,3,4-tetrahydro-1-methyl-β-carboline-3-carboxylic acid (**12**) [24,25] and Petroselenic acid (**13**), were also dereplicated in the DCM fraction of *C. siphonella*. Furthermore, Petroselinic acid (**13**) was reported to have a considerable antimicrobial activity against several bacteria, yeast, and mold species [26].

### 2.2. Assessment of Antimicrobial Activity

For antibacterial testing, the crude methanolic extract, all fractions were initially evaluated in vitro against *Bacillus subtilis* (ATCC 5230), *Staphylococcus aureus* (ATCC 25923), *Pseudomonas aeruginosa* (ATCC 9027), and *Escherichia coli* (ATCC 25922). The antibacterial activities were recorded as inhibition zone diameter and measured on the basis of ‘mm’ (Table 3). Ampicillin and gentamicin were used as positive control. *Staphylococcus aureus* (6.6 mm), *Bacillus subtilis* (5.4 mm), and *Escherichia coli* (1.5 mm) were the three bacteria that were most inhibited by DCM fraction, while hexane fraction only displayed mild antibacterial activity with inhibition zones ranging from 1–2 mm. Furthermore, DCM fraction and its purified metabolites were then evaluated for their antimicrobial activity against various Gram-positive and Gram-negative bacterial strains, yeast, and fungi as mentioned in Section 3.3. According to inhibition ratio percentage calculation (Table 4), all samples displayed no activity against Gram-positive *Salmonella typhi*, and Gram-negative *Klebsiella pneumoniae*, yeast and fungi. In addition, the highest inhibition was observed against *E. coli* (70.50%), concerning compound (**13**) and also against *S. aureus* with inhibition ratio percentage of 60.90 caused by compound (**3**, callysterol) and compound (**11**, siphonellinol C). On the other hand, compound (**11**) individually showed a considerable inhibition against MRSA with an inhibition ratio of 50.374%. Furthermore, the minimum inhibitory concentration (MIC) values of DCM fraction and purified metabolites that showed antibiotic activities were determined (Table 5). Compounds, (**3**) and (**11**) possessed the lowest MIC against *S. aureus* (6.25 µg/mL), compared to ciprofloxacin positive control (1.25 µg/mL), suggesting the make use of those metabolites as future drug leads.

### 2.3. Structure Characterization of the Purified Metabolites

Three known sipholanes, sipholenol A (**1**), sipholenone A (**2**), and siphonellinol C (**11**) [13,27] along with one steroidal compound, callysterol (**3**) [9], and one high intensity fatty acid, namely petroselenic acid (**13**) [17,18], were also isolated from the DCM fraction of *C. siphonella* (Figure 1). Based on accurate mass analyses and comparisons of their NMR spectroscopic data with those reported in the literature, all of those metabolites were identified (Figures S1–S5 and Tables S1–S5, Supplementary Materials).

### 2.4. Biofilm Inhibitory Activity

**Figure 2:**
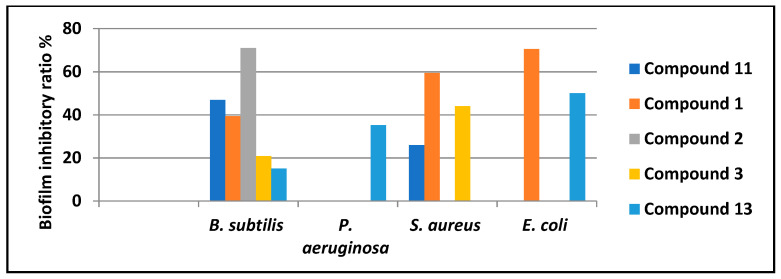
Biofilm inhibitory ratio of DCM Fraction purified metabolites. Compound numbers are the same as those presented in Table 1 and Table 2.

As demonstrated from the antimicrobial screening, metabolites (**3**) and (**11**), and (**13**) exhibit a considerable inhibition against some bacterial strains. As a result, those compounds may have the potential to inhibit biofilm formation. According to a recent analysis [28], numerous categories of natural compounds, such as metabolites originating from marine invertebrates, can prevent the growth of biofilms and are hence suitable for adjuvant therapy as supplements to the standard antibiotics. Therefore, DCM fraction and isolated compounds were tested for their anti-biofilm activities in *Bacillus subtilis* (ATCC 5230), *Staphylococcus aureus* (ATCC 25923), *Pseudomonas aeruginosa* (ATCC 9027), and *Escherichia coli* (ATCC 25922) and each of these bacteria’s biofilms was compared to the control (untreated biofilms). As a result, compound (**1**) exhibited a powerful biofilm inhibitory activity in *S. aureus* and *E. coli*, while compound (**2**) displayed a significant inhibition in *B. subtilis* (Figure 2). To the best of our knowledge, none of the derivatives of sipholane have been linked to a reduction in the development of biofilms by human pathogens. Therefore, additional mechanistic research on these compounds is required.

### 2.5. Docking Study

In order to get insight into the possible target and mode of action of compound **11**, which showed the highest antibacterial activity, the modeled structure of compound **11** was subjected to a pharmacophore-based virtual screening using Swiss-Similarity online-software (http://www.swisssimilarity.ch/, accessed on 1 November 2022) [28]. The retrieved results showed that compound **11** matching the pharmacophore features of the well-known antibacterial agent fusidic acid with a good score of 0.576. Accordingly, it can be concluded that compound **11** may exert its antibacterial effect via targeting the same molecular target of fusidic acid, the elongation factor G that has an essential role of peptide eleongation during protein synthesis by ribosomes [29,30]. Hence, we downloaded and prepared the previously characterized crystal structure of EF-G in complex with fusidic acid [31] to use it in docking of compound **11**. As a validation step of the docking protocol, fusidic acid was re-docked into the binding site of the elongation factor G using Autodock vina. The generated binding pose was very similar to that co-crystalized one with RMSD (the root mean square difference) value of 1.35 Å. The predicted binding affinity scores of both fusidic acid and compound **11** were −9.78 and −9.21 kcal/mol.

**Figure 3:**
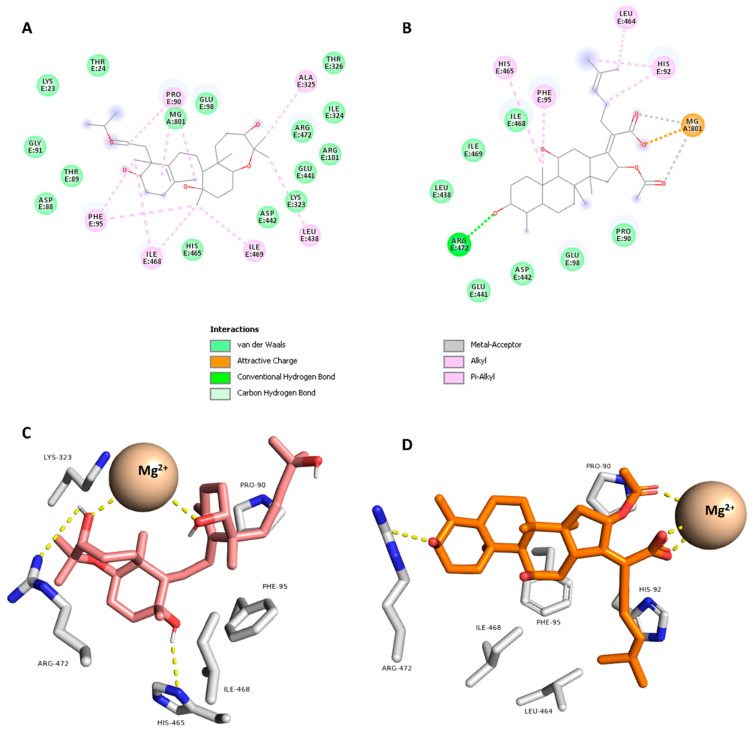
Binding mode of compound **11** and fusidic acid inside the binding site of EF-G (PDB ID: 7N2C) using 2D (**A** and **B**, respectively) and 3D representations (**C** and **D**, respectively).

As shown in Figure 3, compound **11** established hydrophilic and hydrophobic interactions similar to that of the co-crystalized inhibitor fusidic acid. For instance, it formed H-bonds with ARG-472 and HIS-469 in addition to four hydrophobic interactions with PRO-90, PHE-95, LYS-323, and ILE-468. Taken together, compound **11** is a promising scaffold for future development of new antibacterial compounds targeting EF-G. It is worth noting that the predicted target (i.e., EF-G) is an *E. coli* protein, however, this target has high similarity with those of *B. subtilis*, *P. aeruginosa* and *S. aureus*, particularly the conserved fusidic acid binding site [32]. Hence, EF-G (PDB ID: 7N2C) is a good model for other bacterial species tested in the present study.

## 3. Materials and Methods

### 3.1. Sponge Material

The marine sponge *C. siphonella* (1 kg) was discovered in February 2022 at a depth of 9 m off the coast of Hurghada in the Red Sea (27°15048″ north (N), 33°4903″ east (E). At the Invertebrates Department of the National Institute of Oceanography and Fisheries, Red Sea Branch, Hurghada, Egypt, a voucher sample (NIOF209/2022) was reserved.

### 3.2. Extraction and Fractionation

Small chunks of the frozen sample of sponge material (1 kg fresh weight) were sliced and extracted with methanol using an ultrasonic device (4 × 500 mL). A rotary evaporator (Buchi, Flawil, Switzerland) was used to condense the resulting liquid extract. The concentrated extract (semisolid brown residue, 35 g) was divided using modified Kupchan’s solvent partition method (Kupchan et al., 1973) between *n*-hexane (3 × 200 mL), dichloromethane (4 × 200 mL), and n-butanol after being suspended in distilled water (2 × 200 mL). After being independently concentrated under reduced pressure, each fraction was tested for its antibacterial and anti-biofilm properties.

### 3.3. Assessment of Antimicrobial Activity

According to El-Ghorab et al. [33], the antimicrobial activity of MeOH extract and their fractions, as well as DCM fraction purified compounds were tested, respectively. The methods were detailed in Section S6 of the “Supplementary Material File”.

### 3.4. Metabolomics Analysis

On a Synapt G2 HDMS quadrupole time-of-flight hybrid mass spectrometer connected to an Acquity Ultra Performance Liquid Chromatography system (Waters, Milford, CT, USA), metabolomic profiling was carried out on the most powerful fraction (dichloromethane) of *C. siphonella*. Chromatographic separation was performed on a BEH C18 column (2.1 × 100 mm, 1.7 m particle size; Waters, Milford, CT, USA) with a guard column (2.1 × 5 mm, 1.7 m particle size) using 0.1% formic acid in water (*v*/*v*) as solvent A and acetonitrile as solvent B over 6 min at a flow rate of 0.3 mL.min^−1^. The column temperature was 40 °C, and the injection volume was 2 µL. The raw data were transformed using MS Converter software into 2 separate files for positive and negative ionization. Data mining software MZmine 2.10 (Okinawa Institute of Science and Technology Graduate University, Japan) was then used to process the obtained files for deconvolution, peak picking, alignment, deisotoping, and formula prediction. The databases used for the identification of compounds were: Dictionary of Natural Products on DVD (DNP) 2020, and MarinLit: http://pubs.rsc.org/marinlit/, accessed on 1 November 2022. 

### 3.5. Isolation and Purification

The gradient elution method was used to further fractionate the active DCM fraction (10 g) on a silica gel column, yielding four sub fractions (codes F1–F4). Compounds [**3** (30 mg) and **1** (20 mg)], were obtained by chromatographic separation of sub fraction F1 on a silica gel column using n-hexane/ethylacetate in a gradient elution method. Compounds [**2** (25 mg) and **13** (15 mg)], were obtained by chromatographing sub fraction F2 on a silica gel column using n-hexane/ethylacetate in a gradient elution. Additionally, sub fractions F3–4 were mixed, chromatographed on a silica gel column utilizing a gradient elution of DCM and MeOH, and this produced compound **11** (10 mg).

### 3.6. Biofilm Inhibitory Activity

Using a microtiter plate assay (MTP) in 96 wells of flat-bottom polystyrene titre plates, the biofilm inhibitory activity of the obtained extracts various fractions was evaluated against four clinical microorganisms (*P. aeruginosa*, *S. aureus*, *E. coli*, and *B. subtilis*) (Hamed et al., 2020). Briefly, 180 µL of LB broth (tryptone 10 g, yeast extract 5 g, NaCl 10 g/L), 10 µL of an overnight pathogenic bacterial culture, and 10 µL of the tested fractions were added to each well, and they were then incubated at 37 °C for 24 h versus a blank control. Following incubation, the contents of the wells were removed, and free-floating bacteria were eliminated by washing them with 200 µL of phosphate buffer saline (PBS), pH 7.2. 2%. Sodium acetate and 0.1% crystal violet were used to fix and stain the sessile bacteria’s adhesion, respectively. Extra stain was washed with deionized water and left to dry. A microtitre plate reader (BMG LABTECH GmbH, Allmendgrün, Germany) was used to measure optical density (OD) at 595 nm after dried plates had been cleaned with 95% ethanol.

### 3.7. Docking Study

AutoDock Vina software was used for the docking study [34]. Compound **11** was drawn and prepared to be docked into the binding site of EF-G. (PDB codes: 7N2C) [31]. The binding site was determined according to the enzyme’s co-crystallized ligands (FUA). The co-ordinates of the grid boxes were: x = 283.653; y = 246.548; z = 289.228. The size of the grid box was set to 20 Å. Exhaustiveness was set to 24. Ten poses were generated for each docking experiment. Docking poses were analyzed and visualized using Pymol and Biovia software [34].

## 4. Conclusions

The current study revealed the marine sponge *C. siphonella*’s antimicrobial and anti-biofilm properties concerning methanolic extract and its various fractions, as well as metabolites purified from DCM fraction, the most bioactive one. Furthermore, chemical profile of *C. siphonella* DCM fraction demonstrated its capacity to assemble and produce a number of secondary metabolites, primarily sipholanes, implying their involvement in *C. siphonella*’s previously reported anticancer activities. Because of the combined effects of these phytochemicals and/or their synergistic interactions, the antibacterial activity of the *C. siphonella* DCM fraction may be partially attributed to these factors. The antibacterial study confirms that the compound **11**, namely siphonellinol C is the most effective metabolite at controlling the development of the microorganisms tested particularly *S. aureus* and MRSA that can be assured by calculation of its MIC. In accordance with docking study, the higher antibacterial activity of siphonellinol C, could be caused by targeting elongation factor G, the same molecular target as fusidic acid, which is vital for peptide elongation during protein biosynthesis by bacterial ribosomes. These new findings might help sipholanes’ therapeutic potential in the future. Given their high concentrations and reported activity, sipholanes may be thought of as offering protection from a number of disorders. More investigation into the cellular processes and molecular components of sipholanes’ antibacterial and anti-biofilm properties will soon be needed.

## Figures and Tables

**Table 1:** Dereplication of the metabolites identified from *C. siphonella* DCM fraction.

NP	Tentative Identification ^b^	Quasi-Form	Suggested Formula ^a^	Calculated Accurate *m*/*z*	Experimentally Accurate *m*/*z*
1	Sipholenol A	[M+H]^+^	C_30_H_53_O_4_	477.3944	477.3941
2	Sipholenone A	[M+H]^+^	C_30_H_51_O_4_	475.3785	475.3787
3	Callysterol	[M+H]^+^	C_28_H_49_O	401.381	401.3783
4	Cholestenone	[M+H]^+^	C_27_H_45_O	385.3472	385.347
5	5α-cholestanone	[M+H]^+^	C_27_H_47_O	387.3625	387.3627
6	Stigmasterone	[M+H]^+^	C_29_H_47_O	411.3634	411.3637
7	stigmasta-4,22-dien-3,6-dione	[M+H]^+^	C_29_H_45_O_2_	425.345	425.342
8	Sipholenoside B	[M+H]^+^	C_36_H_63_O_8_	623.452	623.4523
9	Sipholenol G	[M+H]^+^	C_30_H_53_O_5_	493.3889	493.3893
10	Sipholenoside A	[M+H]^+^	C_36_H_61_O_8_	621.4368	621.4366
11	Siphonellinol C	[M+H]^+^	C_30_H_52_O_5_	492.3814	492.3820
12	1,2,3,4-tetrahydro-1-methyl-β-carboline-3-carboxylic acid	[M+H]^+^	C_13_H_15_N_2_O_2_	231.1133	231.1134
13	Petroselenic acid	[M+H]^+^	C_18_H_35_O_2_	283.2634	283.2637

^a^ High-resolution electrospray ionization mass spectrometry (HRESIMS) using XCalibur 3.0 and allowing for M+H/M+Na adduct. ^b^ The suggested compound according to the Dictionary of Natural Products (DNP 23.1, 2021 on DVD) and Reaxys online database.

**Table 2:** High features of compounds (ranked by peak intensity) detected in DCM fraction of *C. siphonella* after dereplication of its metabolomes.

NP	Tentative Identification ^b^	Intensity	Suggested Formula ^a^	Calculated Accurate *m*/*z*	Experimentally Accurate *m*/*z*
1	Sipholenol A	2.2 × 10^4^	C_30_H_53_O_4_	477.3944	477.3941
2	Sipholenone A	1.2 × 10^7^	C_30_H_51_O_4_	475.3785	475.3787
3	Callysterol	4.4 × 10^7^	C_28_H_49_O	401.381	401.3783
11	Siphonellinol C	8.8 × 10^5^	C_30_H_52_O_5_	492.3814	492.3820
13	Petroselenic acid	2.3 × 10^7^	C_18_H_35_O_2_	283.2634	283.2637

^a^ High-resolution electrospray ionization mass spectrometry (HRESIMS) using XCalibur 3.0 and allowing for M+H/M+Na adduct. ^b^ The suggested compound according to the Dictionary of Natural Products (DNP 23.1, 2021 on DVD) and Reaxys online database.

**Table 3:** Inhibition zone diameter (mm) of the methanol extract and fractions of *C. siphonella* on *S. aureus*, *B. subtilis*, *E. coli* and *P. aeruginosa* (Mean ± S.E).

Tested Extract	*S. aureus*	*B. subtilis*	*E. coli*	*P. aeruginosa*
MeOH Ext	1.1 ± 0.5	1.2 ± 0.2	-	-
Hex Fr	2.3 ± 0.9	1.1 ± 0.4	-	1 ± 0.4
DCM Fr	6.6 ± 0.2	5.4 ± 0.3	1.5 ± 0.7	-
ButOH Fr	-	0.5 ± 0.2	-	-
Ampicillin	13.7 ± 0.9	12.3 ± 1.2	3.9 ± 0.9	3.6 ± 0.3
Gentamicin	9.8 ± 1.2	10.1 ± 1.1	15.5 ± 0.1	14.8 ± 1.3

Ampicillin, gentamicin, extracts and fractions (20 μg/mL DMSO). EtOH, ethanol extract; Hex Fr, *n*-hexane fraction; DCM Fr, dichloromethane fraction; ButOH Fr, *n*-butanol fraction.

**Table 4:** In vitro antimicrobial activity of DCM Fraction purified metabolites.

	Inhibition Ratio (%)						
	Kle	Sal	Sta	MRSA	Ech	Can	Asp
11 *	NA	NA	60.90	50.374	NA	NA	NA
1 *	NA	NA	35.216	NA	45.50	NA	NA
2 *	NA	NA	55.92	40.30	50.0	NA	NA
3 *	NA	NA	60.90	NA	NA	NA	NA
13 *	NA	NA	30.00	NA	70.50	NA	NA
Nys	-	-	-	-	-	97	98
Cip	98	-	96	-	98	-	-

Ciprofloxacin, nystatin, and isolated compounds (20 μg/mL DMSO). Nys, nystatin; Cip, ciprofloxacin; Kle, *K. pneumoniae*; Sal, *S. typhi*; Sta, *S. aureus*; MRSA, Methicillin-resistant *S. aureus*; Ech, *E. coli*; Can, *Candida albicans*; Asp, *Aspergillus niger*. *: Compound numbers are the same as those presented in Table 1 and Table 2.

**Table 5:** Minimum inhibitory concentration (MIC) of DCM Fr purified metabolites.

	MIC (µg/mL)		
	*S. aureus*	MRSA	*E. coli*
11 *	6.25	12.50	NA
1 *	12.5	NA	25.00
2 *	12.5	25.00	NA
3 *	6.25	NA	NA
13 *	25.00	NA	NA
Cip	1.25	-	0.390

Ciprofloxacin, nystatin, DCM Fraction isolated compounds (dissolved in DMSO). Cip, ciprofloxacin; MRSA, Methicillin-resistant *S. aureus*. *: Compound numbers are the same as those presented in Table 1 and Table 2.

## Supplementary Materials

The following supporting information can be downloaded at: https://www.mdpi.com/article/10.3390/antibiotics11121682/s1, Figure S1: ^1^H (400 MHz) NMR spectroscopic data for compound **3** in CDCl_3_; Table S1: ^1^H (400 MHz) NMR spectroscopic data for compound **3** in CDCl_3_; Figure S2: ^1^H (400 MHz) NMR spectroscopic data for compound **1** in CDCl_3_; Table S2: ^1^H (400 MHz) NMR spectroscopic data for compound **1** in CDCl_3_; Figure S3: ^1^H (400 MHz) NMR spectroscopic data for compound **2** in CDCl_3_; Table S3: ^1^H (400 MHz) NMR spectroscopic data for compound **2** in CDCl_3_; Figure S4: ^1^H (400 MHz) NMR spectroscopic data for compound **13** in CDCl_3_; Table S4: ^1^H (400 MHz) NMR spectroscopic data for compound **13** in CDCl_3_; Figure S5: ^1^H (400 MHz) NMR spectroscopic data for compound **11** in MeOD; Table S5: ^1^H (400 MHz) NMR spectroscopic data for compound **11** in MeOD; Section S1: Identification and Structural elucidation of compound **3**, callysterol; Section S2: Identification and Structural elucidation of compound **1**, sipholenol; Section S3: Identification and Structural elucidation of compound **2**, sipholenone; Section S4: Identification and Structural elucidation of compound **13**, Petroselenic aci; Section S5: Identification and Structural elucidation of compound **11**, Siphonellinol C; Section S6. Assessment of antimicrobial activity. Reference [35] is cited in the Supplementary Materials.
