# Supplementary material for: Screening and Molecular Docking of Bioactive Metabolites of the Red Sea Sponge Callyspongia siphonella as Potential Antimicrobial Agents
Source: Antibiotics (Basel). 2022 Nov 23;11(12):1682. doi: 10.3390/antibiotics11121682 (PMC9774121; doi:10.3390/antibiotics11121682)
Supplement: Supplementary file 1 [file antibiotics-11-01682-s001.zip › antibiotics-2035405-supplementary.pdf]

Supplementary Material for *Antibiotics*

Screening and Molecular Docking of Bioactive Metabolites of the Red Sea Sponge *Callyspongia siphonella* as Potential Antimicrobial Agents

Arafa Musa [1,*], Mohamed A. Abdelgawad [2,*], Mohamed E. Shaker [3], Ahmed H. El-Ghorab [4], Della Grace Thomas Parambi [2], Ahmed A. Hamed [5], Ahmed M. Sayed [6], Hossam M. Hassan [7] and Mahmoud A. Aboseada [6]

[1] Department of Pharmacognosy, College of Pharmacy, Jouf University, Sakaka 72341, Aljouf, Saudi Arabia
[2] Department of Pharmaceutical Chemistry, College of Pharmacy, Jouf University, Sakaka 72341, Aljouf, Saudi Arabia
[3] Department of Pharmacology, College of Pharmacy, Jouf University, Sakaka 72341, Aljouf, Saudi Arabia
[4] Department of Chemistry, College of Science, Jouf University, Sakaka 72341, Aljouf, Saudi Arabia
[5] National Research Centre, Microbial Chemistry Department, 33 El-Buhouth Street, Dokki, Giza 12622, Egypt
[6] Department of Pharmacognosy, Faculty of Pharmacy, Nahda University, Beni-Suef 62513, Egypt
[7] Department of Pharmacognosy, Faculty of Pharmacy, Beni-Suef University, Beni-Suef 62513, Egypt
* Correspondence: akmusa@ju.edu.sa (A.M.); mhmdgwd@ju.edu.sa (M.A.A.)

Abstract: Marine sponges create a wide range of bioactive secondary metabolites, as documented throughout the year. Several bioactive secondary metabolites were isolated from different members of *Callyspongia siphonella* species. This study aimed for isolation and structural elucidation of major metabolites in order to investigate their diverse bioactivities such as antimicrobial and anti-biofilm activities. Afterwards, molecular docking study was conducted, searching for the possible mechanistic pathway of the most bioactive metabolites. Extraction, fractionation, and metabolomics analysis of different fractions was performed in order to obtain complete chemical profile. Moreover, in vitro assessment of different bioactivities was done, using recent techniques. Additionally, purification, structural elucidation of high features using recent chromatographic and spectroscopic techniques was established. Finally, AutoDock Vina software was used for the Pharmacophore-based docking-based analysis. As a result, DCM fraction exerted the best antibacterial activity using disc diffusion method; particularly against *S. aureus* with an inhibition zone of 6.6 mm. Compound 11 displayed a considerable activity against both MRSA and *S. aureus* with inhibition ratios of 50.37 and 60.90%, respectively. Concerning anti-biofilm activity, compounds 1 and 2 displayed powerful activity with inhibition ratios ranging from 39.37% to 70.98%. Pharmacophore-based docking-based analysis suggested elongation factor G (EF-G) to be a probable target for compound 11 (siphonellinol C) that showed the best in vitro antibacterial activity, offering unexplored potential for new drugs and treatment candidates.

S1. Identification and Structural elucidation of compound (3), callysterol

Compound **3** was isolated as white crystals (30 mg); ESI-MS analysis resulted in *m/z* 399.35 corresponding to [M-1]- ; for complete NMR data see (Table S1). The ^{1}H NMR (Figure S1) showed characteristic methyl groups at δH 0.67 (s), 1.16 (s), 0.95 (d), 0.8 (d), indicating the presence of cholestan structure. The resonance at δH 5.28 (br s) indicated the presence of double bond.. According to these data, ESI-MS analysis and previously published data [1], compound **3** was identified as callysterol, a known compound which isolated before from *Callyspongia siphonella*.

Table S1. ^{1}H (400 MHz) NMR spectroscopic data for compounds 3 in $CDCl_3$.

Position	δH, mult. (*J* in Hz)	Type
1	1.76 (m)	CH2
2	1.37 (m), 1.36 (m)	CH2
3	3.49 (m)	C
4	2.26 – 2.21 (m)	CH
6	5.28 (br s)	C
7	1.93 (m)	CH_2
8	1.37 (m), 1.36 (m)	CH
9	0.93 (m)	CH
11	5.16 (m)	CH_2
12	5.15 (d)	CH_2
14	1.82 (m)	CH
15	0.89 (m)	CH_2
16	1.12 (m), 1.07 (m)	CH_2
17	1.87 (m)	CH
18	0.99 (m)	CH_3
19	0.91 (m)	CH_3
20	1.15 (m)	CH
21	0.67 (d, 2.4)	CH_3
22	1.17 (m), 1.14 (m)	CH
23	0.97 (m)	CH
24	1.13 (m)	CH_2
25	1.67 (m)	CH
26	0.80 (d, 6.1)	CH_3
27	0.80 (d, 6.1)	CH_3
28	0.85 (d, 6.5)	CH_2

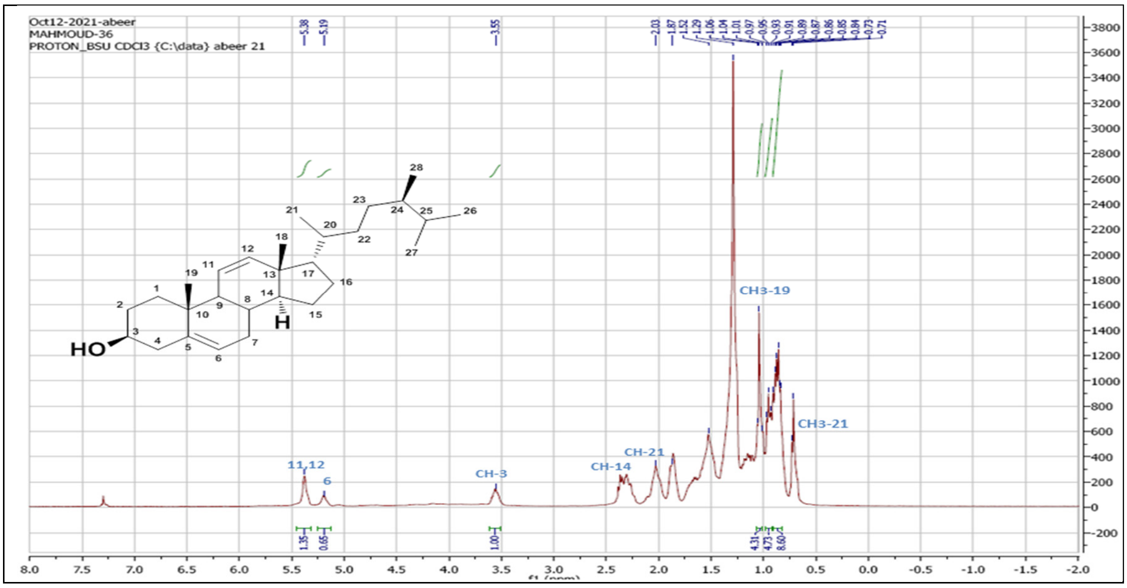

Figure S1. ^{1}H (400 MHz) NMR spectroscopic data for compounds 3 in $CDCl_3$.

S2. Identification and Structural elucidation of compound (1), sipholenol

Compound **1** was isolated as colorless crystals (20 mg); ESI-MS analysis resulted in *m/z* 475.36 corresponding to [M-1]- ; for complete NMR data see (Table S2). The ^{1}H NMR (Figure S2) showed characteristic seven methyl groups at δH 0.92 (s), 0.96 (s), 1.01(s), 1.02 (s), 1.06 (s), 1.18 (s), 1.19 (s), 1.69 (s). The resonance at δH 5.38 (br dd,) indicated the presence of double bond. The structural information from the ^{1}H NMR spectra of compound **1** is quite limited because of the relatively high number of un-functionalized methylene groups and tertiary functional groups. According to these data, ESI-MS analysis and previously published data [2], compound **1** was identified as sipholenol A, a known compound which isolated before from *Callyspongia siphonella*. Sipholenol A is a characteristic triterpene for the Red Sea sponge *Callyspongia siphonella*. It has been previously reported as a potent P-glycoprotein inhibitor in multi drug resistant cancer cells [3].

Table S2. ^{1}H (400 MHz) NMR spectroscopic data for compounds 1 in $CDCl_3$.

Position	δH, mult. (*J* in Hz)	Type
**3**	2.41, (m, 2H)	CH2
**4**	**3.74 (d, 6.8)**	**CH**
**7**	**3.44 (dd, 11,6, 4.4)**	**CH**
**16**	5.39 (br dd, 8, 5,2)	CH
**24**	0.96 (s)	CH3
**25**	1.19 (s)	CH3
**26**	1.14 (s)	CH3
**28**	1.69 (s)	CH3
**29**	**1.06 (s)**	**CH_3**
**30**	**0.96 (s)**	**CH_3**
**31**	1.01 (s)	CH3

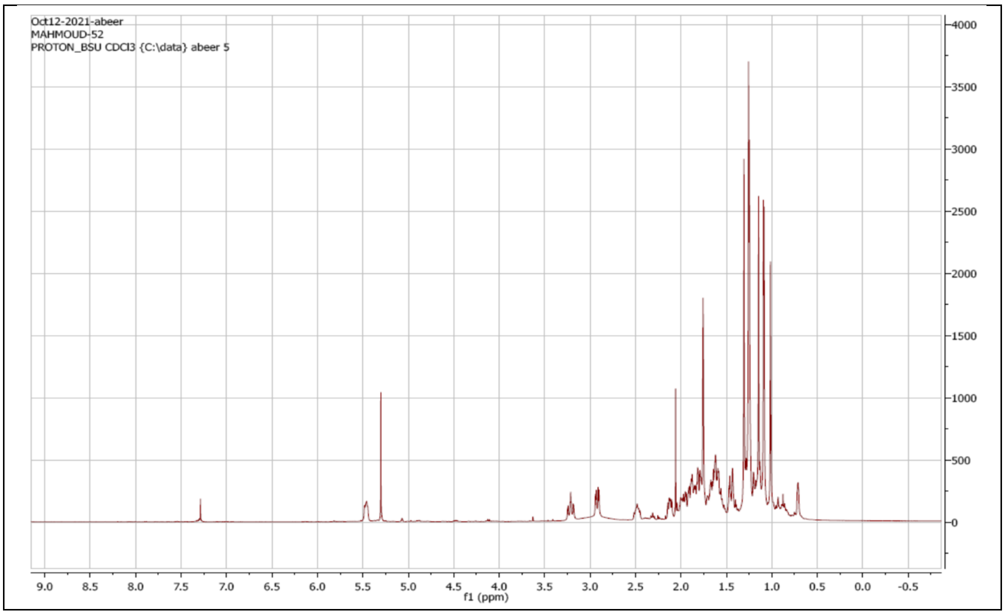

Figure S2. ^{1}H (400 MHz) NMR spectroscopic data for compounds 1 in $CDCl_3$.

S3. Identification and Structural elucidation of compound (2), sipholenone

Compound 2 was isolated as colorless crystals (25 mg); ESI-MS analysis resulted in *m/z* 473.36 corresponding to $[M-1]^-$; for complete NMR data see (Table S3). The ^{1}H NMR (Figure S3) showed characteristic seven methyl groups at δH 0.95 (s), 1.02 (s), 1.08 (s), 1.18 (s), 1.19 (s), 1.24 (s), 1.69 (s). The resonance at δH 5.4 (br dd,) indicated the presence of double bond. The structural information from the ^{1}H NMR spectra of compound **3** is quite limited because of the relatively high number of un-functionalized methylene groups and tertiary functional groups. According to these data, ESI-MS analysis and previously published data [2], compound **2** were identified as sipholenone A, a known compound which isolated before from *Callyspongia siphonella*. Sipholenone A is a characteristic triterpene for the Red Sea sponge *Callyspongia siphonella*. It has been previously reported as a potent P-glycoprotein inhibitor in multi drug resistant cancer cells [3].

Table S3. ^{1}H (400 MHz) NMR spectroscopic data for compounds 2 in $CDCl_3$.

Position	δH, mult. (*J* in Hz)	Type
3	2.05 (ddd), 3.18 (ddd)	CH2
7	2.85 (dd, 11.6, 4)	CH
16	5.4 (br dd)	CH
17	2.42, (m, 1H)	CH2
24	0.95 (s)	CH3
25	1.24 (s)	CH3
26	1.19 (s)	CH3
27	1.18 (s)	CH3
28	1.7 (s)	CH3
29	1.09 (s)	CH3
30	1.02 (s)	CH3
31	1.02 (s)	CH3

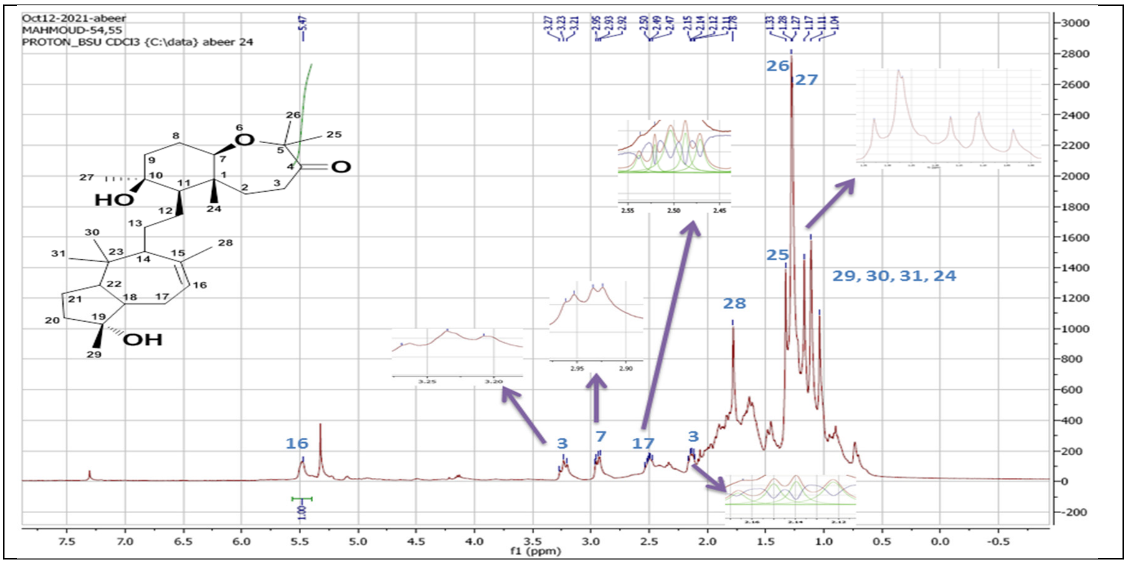

Figure S3. ^{1}H (400 MHz) NMR spectroscopic data for compounds 2 in $CDCl_3$.

S4. Identification and Structural elucidation of compound (13), Petroselenic acid

Compound **13** was isolated as white powder (15 mg); ESI-MS analysis resulted in *m/z* 281.25 corresponding to $[M-1]^-$; for complete NMR data see (Table S4). The ^1H NMR (Figure S4) data of compound **13** showed characteristic resonances for olefinic protons at *δ*H 5.33, 5.37 with *cis* configuration (*J* = 8 Hz), nine aliphatic moieties at *δ*H 1.29 – 1.33 (m) and a methyl moiety at *δ*H 0.90 (t). According to these data, ESI-MS analysis and previously published data [4], compound **13** identified as petroselinic acid, a known compound which previously isolated from *Callyspongia siphonella*. Petroselinic acid is found in high amounts in the seed oils from plants belonging to the *Apiaceae* family, also known as *Umbelliferae,* and the *Araliaceae* family. Petroselinic acid has a considerable antimicrobial activity against several bacteria, yeast and mold species [5].

Table S4. ^1H (400 MHz) NMR spectroscopic data for compounds 13 in $CDCl_3$.

Position	δH, mult. (*J* in Hz)	Type
1	0.90, (t, 3H)	CH3
2	1.29 – 1.33, (m, 2H)	CH2
3	1.29 – 1.33, (m, 2H)	CH2
4	1.29 – 1.33, (m, 2H)	CH
5	1.29 – 1.33, (m, 2H)	CH2
6	1.29 – 1.33, (m, 2H)	CH2
7	1.29 – 1.33, (m, 2H)	CH2
8	1.29 – 1.33, (m, 2H)	CH2
9	1.29 – 1.33, (m, 2H)	CH2
10	1.29 – 1.33, (m, 2H)	CH2
11	2.03, (dd, J = 6 Hz, 4 Hz, 2H)	CH2
12	5.33, (dd, J = 8 Hz, 4 Hz, 1H)	CH
13	5.37, (dd, J = 8 Hz, 4 Hz, 1H)	CH
14	2.06, (dd, J = 6 Hz, 4 Hz, 2H)	CH2
15	1.38, (p, 2H)	CH2
16	1.61, (p, 2H)	CH2
17	2.15, (t, 2H)	CH2

S5. Identification and Structural elucidation of compound (11), Siphonellinol C

The HREIMS data of siphonellinol C (**11**) showed a molecular ion peak at *m/z* 515.3708 $[M - 1]^-$ corresponding to the molecular formula $C_{30}H_{52}O_5$ and suggested five degrees of unsaturation. The ^1H NMR data (Table S5) showed the presence of two double bonds, indicating three rings in the structure. The methyl singlet at ***δ*** 0.98 was assigned to H3-26. The methyl singlets at ***δ*** 1.26 and ***δ*** 1.12 were assigned to H3-27 and H3-28, respectively. The methyl singlet at ***δ*** 1.23 was assigned H3-29. The right-hand section of the molecule was found to possess a six-membered ring, C, with an attached six-carbon chain. The methine proton at ***δ*** 3.60 (dd, *J*) 9.6, 2.9 Hz) was assigned to H-18,. Olefinic protons resonating at ***δ*** 5.46 (ddd, 15.8, 8.1, 6.2 Hz) and 5.63 (d, 15.8 Hz) were assigned as H-21 and H-22, respectively. Two methyl singlets at ***δ*** 1.28 were assigned as (H3- 24 and H3-25). The stereochemistry of the $\Delta^{21,22}$-system was assigned *E* on the basis of large coupling constant (15.8 Hz) of H-21 and H-22. The splitting pattern and *J* values of proton H-18 indicated its

pseudoaxial orientation. A modeling study of ring C suggested that the pseudoaxially oriented substituent should be in the α-orientation.

Table S5. ^{1}H (400 MHz) NMR spectroscopic data for compound 11 in MeOD.

Position	δH, mult. (*J* in Hz)	Type
**2**	**1.38, 1.52, m**	**CH2**
**3**	**1.74, 1.98, m**	**CH2**
**4**	**3.81, d (6.6)**	**CH**
**6**	**3.52, dd (12.1, 4.4)**	**CH**
**7**	**1.38, 1.71, m**	**CH2**
**9**	**1.60, m**	**CH2**
**11**	**0.88, m**	**CH**
**12**	**1.46, m**	**CH2**
**13**	**2.04, m**	**CH2**
**16**	**2.02, m**	**CH2**
**17**	**1.70, m**	**CH2**
18	3.60, dd (9.6, 2.9)	CH
**20**	**2.13, dd (14.6, 6.2); 2.27, dd (14.6, 8.1)**	**CH2**
**21**	**5.46, ddd (15.8, 8.1, 6.2)**	**CH**
**22**	**5.63, d (15.8)**	**CH**
**24**	**1.28, s**	**CH3**
**25**	**1.28, s**	**CH3**
**26**	**0.98, s**	**CH3**
**27**	**1.26, s**	**CH3**
**28**	**1.12, s**	**CH3**
**29**	**1.23, s**	**CH3**
30	1.67, s	CH3
**31,**	**21.1**	**CH3**

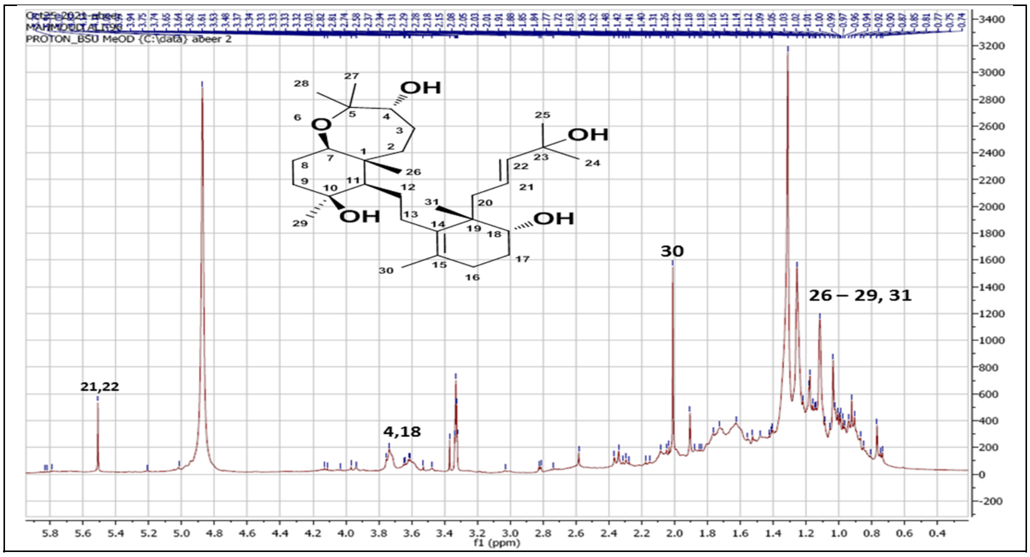

Figure S5. ^{1}H (400 MHz) NMR spectroscopic data for compounds 11 in MeOD.

S6. Assessment of antimicrobial activity

Gram-positive bacteria (*Staphylococcus aureus*, Methicillin-resistant *Staphylococcus aureus* (MRSA)), yeast (*Candida albicans*), fungi (*Aspergillus niger*), and Gram-negative bacteria (*Escherichia coli*, *Pseudomonas aeruginosa*, *Salmonella typhi*, and *Klebsiella pneumoniae*) were used as test organisms to determine the antibacterial activity of DCM fraction and its purified metabolites [6]. The experiment was carried out in 96-well flat polystyrene plates. Following the addition of 10 μL of test extracts (final concentration of 250 μg/mL) to 80 μL of lysogeny broth (LB broth) and 10 μL of bacterial culture suspension (log phase), the plates were incubated at 37 °C for an overnight period. After incubation, the tested compound's positive antibacterial action was visible as clearance in the wells, whereas for others that had no impact on the bacteria, the growth media in the wells appeared opaque. The pathogen is left untreated as the control. After approximately 20 hours, the absorbance was measured at OD600 in a Spectrostar Nano Microplate Reader (BMG LABTECH GmbH, Allmendgrun, Germany).

References

1. Youssef, D.T.A.; Ibrahim, A.K.; Khalifa, S.I.; Mesbah, M.K.; Mayer, A.M.S.; van Soest, R.W.M. New antiinflammatory sterols from the Red Sea sponges Scalarispongia aqabaensis and Callyspongia siphonella. *Nat. Prod. Commun.* **2010**, *5*, 1934578X1000500107.

2. Shmueli, U.; Carmely, S.; Groweiss, A.; Kashman, Y. Sipholenol and sipholenone, two new triterpenes from the marine sponge siphonochalina siphonella (levi). *Tetrahedron Lett.* **1981**, *22*, 709–712.

3. Shi, Z.; Jain, S.; Kim, I.; Peng, X.; Abraham, I.; Youssef, D.T.A.; Fu, L.; El Sayed, K.; Ambudkar, S. V; Chen, Z. Sipholenol A, a marine-derived sipholane triterpene, potently reverses P-glycoprotein (ABCB1)-mediated multidrug resistance in cancer cells. *Cancer Sci.* **2007**, *98*, 1373–1380.

4. Delbeke, E.I.P.; Everaert, J.; Uitterhaegen, E.; Verweire, S.; Verlee, A.; Talou, T.; Soetaert, W.; Van Bogaert, I.N.A.; Stevens, C.V. Petroselinic acid purification and its use for the fermentation of new sophorolipids. *AMB Express* **2016**, *6*, 28.

5. Placek, L.L. A review on petroselinic acid and its derivatives. *J. Am. Oil Chem. Soc.* **1963**, *40*, 319–329.

6. El-Ghorab, A.H.; Behery, F.A.; Abdelgawad, M.A.; Alsohaimi, I.H.; Musa, A.; Mostafa, E.M.; Altaleb, H.A.; Althobaiti, I.O.; Hamza, M.; Elkomy, M.H. LC/MS Profiling and Gold Nanoparticle Formulation of Major Metabolites from Origanum majorana as Antibacterial and Antioxidant Potentialities. *Plants* **2022**, *11*, 1871.
